# Causal gene identification using mitochondria-associated genome-wide mendelian randomization in atrial fibrillation

**DOI:** 10.3389/fphar.2024.1439816

**Published:** 2024-07-29

**Authors:** Ying Chen, Bingxun Li, Hongxuan Xu, Lin Wu

**Affiliations:** ^1^ Department of Cardiology, Peking University First Hospital, Beijing, China; ^2^ Key Laboratory of Medical Electrophysiology of the Ministry of Education and Institute of Cardiovascular Research, Southwest Medical University, Luzhou, China; ^3^ State Key Laboratory of Vascular Homeostasis and Remodeling, Peking University, Beijing, China

**Keywords:** atrial fibrillation, mitochondria, gene expression, mendelian randomization, methylation

## Abstract

**Background:** Mitochondrial dysfunction is one of the important patho-mechanisms in the development of atrial fibrillation (AF) with underidentified genetic pathophysiology.

**Methods:** Summarized data of methylation, expression and protein abundance levels of mitochondria-related genes were obtained from corresponding studies, respectively. Genes related to mitochondria dysfunction in associations with AF were obtained from the UK Biobank (discovery), and the FinnGen study (replication). Summary-data-based Mendelian randomization analysis (SMR) was performed to assess potential causal relationships between mitochondria-related genes related to the molecular features of AF. Colocalization analysis was further conducted to assess whether the identified signal pairs shared causal genetic variants.

**Results:** Five mitochondria-related genes were found to have causal effects with AF in the sensitivity and the colocalization analyses. Strong associations with increased risk of AF were identified with increased expression level of 4 mitochondria-related genes, including PCCB (OR 1.09, 95% CI 1.05–1.12; PPH4 = 0.95), COX18 (OR 1.83, 95% CI 1.29–2.60; PPH4 = 0.83), SLC25A15 (OR 1.34, 95% CI 1.14-1.58; PPH4 = 0.85), and STX17 (OR 1.16, 95% CI 1.08–1.24; PPH4 = 0.76). Conversely, genetically predicted higher levels expression of UQCC1 (OR 0.94, 95% CI 0.91–0.97) were associated with decreased risk of AF. After further tissue-specific validation, genetically predicted expression levels of PCCB (OR 1.12, 95%, CI 1.01-1.24, *p* = 0.025) and STX17 (OR 1.13, 95%, CI 1.04-1.23, *p* = 0.006) in atrial appendage were strongly associated with the increased risk of AF.

**Conclusion:** Mitochondria-related genes are involved either positively (PCCB, COX18, SLC25A15 and STX17) or negatively (UQCCI) in the pathogenesis and the development of AF. These candidate genes may serve as targets for potential development of agents in the prevention and treatment of AF.

## Introduction

Atrial fibrillation (AF) is the most common type of sustained cardiac arrhythmia characterized with rapid and irregular atrial contractions, leading to increased risks of death, stroke, heart failure and peripheral embolisms, etc. (Joglar et al., 2024) Treatment strategies of AF are developed with significant limitations because the underlying mechanisms responsible for the initiation and promotion of AF are incompletely understood (Nattel et al., 2021). The heart is a highly energy-demanding organ, which makes cardiomyocytes as one of the most mitochondria-rich cell types. Approximately 40% of the cell volume is composed of mitochondria (Murphy and Liu, 2023). Mitochondria are the essential organelles that regulate cellular energy production, metabolism, proliferation and apoptosis (Brown et al., 2021). In patients with AF with irregularly fast heart rate, cardiac energy demand and mitochondrial function increase greatly.

The role of mitochondrial dysfunction was investigated as a contributor to the AF development (Liu et al., 2021). Dysfunctional mitochondria may serve as a source of numerous free radicals to oxidize various intracellular targets, including kinases, sodium and calcium channels, etc (Peoples et al., 2019; Balderas et al., 2024). These alterations directly impact the excitability and intercellular coupling of cardiomyocytes to create functional reentrant circuits. In patients with AF, oxidative stress and mitochondrial DNA (mtDNA) damage are increased in atrial cardiomyocytes (Menezes Júnior et al., 2023). These mitochondrial abnormalities further disrupt cellular energy metabolism and signal transduction, exacerbate cardiomyocyte stress responses, ultimately promote the occurrence and development of AF. Moreover, calcium (Ca^2+^) handling abnormalities and oxidative stress play pivotal roles in the structural and electrical remodelings of the atrial myocytes, potentially contribute to the pathogenesis of AF (Nattel and Dobrev, 2012; Sagris et al., 2021). Mitochondria are the main producers of cellular adenosine triphosphate (ATP) in cardiac myocytes, and both Ca^2+^ and adenosine diphosphate (ADP) are key regulators of respiratory flux to match the energy supply to the constantly varying demands in the heart (Mason et al., 2020).

The specific mitochondria-related genes and their downstream effects on AF remain elusive. Mendelian randomization (MR) is a method using genetic variants as instrumental variables to explore the potential causal relationships between lifetime risk exposure and outcome (Davies et al., 2018). Compared to observational studies, this method is less susceptible to confounding and reverse causation bias as genetic variants are randomly distributed at conception and cannot be modifiable by the onset of the disease. Genome-wide association studies (GWAS) use genetic associations with traits based on single nucleotide polymorphisms (SNPs) and integrate GWAS data with gene expression and methylation GWAS, thereby identifying expression or methylation quantitative trait loci (eQTL, pQTL, or mQTL) (Zhu et al., 2016; McRae et al., 2018; Ferkingstad et al., 2021). The increasing availability of large-scale GWAS and molecular quantitative trait loci data allows us to explore the causal relationship between the regulation of mitochondria-associated genes and AF in terms of methylation, expression, and protein abundance. MR was utilized to investigate the potential association of mitochondria-related genes methylation, expression, and protein abundance with AF risk in this study.

## Methods

### Study design

**FIGURE 1:**
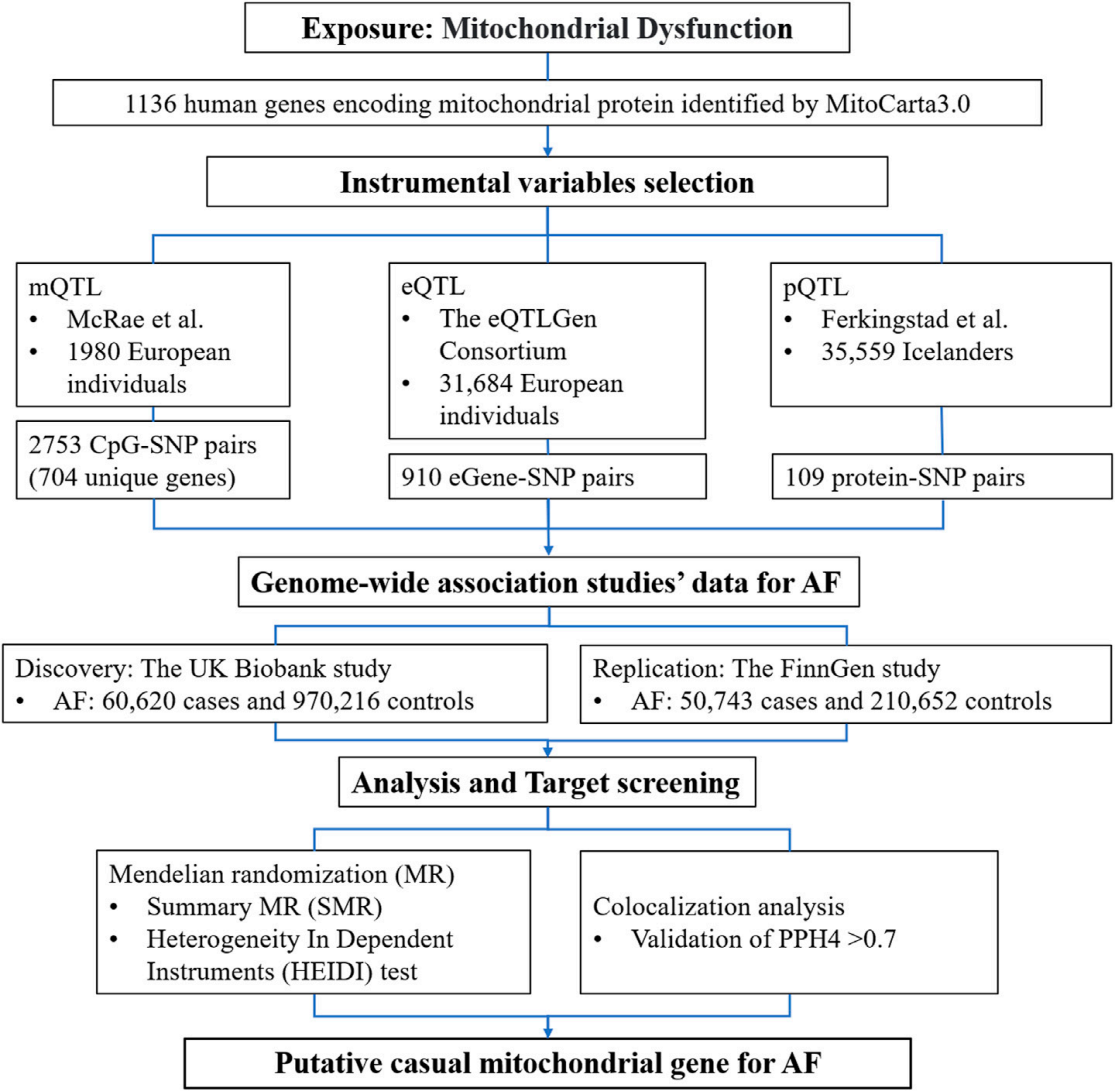
Detailed study design used in this report. SMR, summary-based Mendelian randomization; QTL, quantitative trait loci; AF, atrial fibrillation; SNP, single nucleotide polymorphisms; PPH4, posterior probability of H4.

The study design and the workflow for selected genetic variants and analytical methods were summarized in Figure 1. To determine the mitochondrial dysfunction characterized by the genetic predisposition in the mitochondria-related genome, we extracted a list of 1136 known mitochondria-related genes from the human MitoCarta3.0 database (Rath et al., 2021). The mitochondria-related genes refer to nuclear-encoded mitochondrial proteins, and 13 genes of the mitochondrial genome were not included in this study. Instrumental variables (IVs) for mitochondria-related genes were extracted at the methylation, gene expression, and protein abundance levels. Subsequent MR analyses were conducted for AF at each biological level. Colocalization analyses were then applied to strengthen the causal inference.

### Data sources of methylation, expression, and protein quantitative trait loci

The UK Biobank study served as the primary discovery dataset, with the FinnGen study datasets was utilized for validation and to investigate the gene methylation, gene expression, and protein abundance levels, respectively (Supplementary Table S1). Integration of multi-omic data enabled us to illuminate the underlying molecular networks of mitochondrial dysfunction. QTLs revealed the associations of single nucleotide polymorphisms (SNPs) with levels of DNA methylation, gene expression, and protein abundance. SNP-CpG associations in blood were obtained from mQTL data by McRae et al., in 1980 European ancestry individuals (McRae et al., 2018). Individual methylation probes underwent normalization using a generalized linear model employing a logistic link function, with adjustments made for chip type, sex, age, age squared, sex × age, and sex × age^2^. Blood eQTL data were sourced from the eQTLGen consortium, comprising 31,684 individuals (Võsa et al., 2021). Summary statistics detailing genetic associations with circulating protein levels were obtained from a pQTL study by Ferkingstad et al., involving 35,559 individuals from Iceland (Ferkingstad et al., 2021). Protein levels underwent rank-inverse normal transformation and were adjusted for age, sex, and sample age for each protein analyzed.

The tissue-specific expression of target genes, potential causes of AF, were further evaluated using tissue-specific eQTL data obtained from the Genotype Tissue Expression (GTEx) web portal (https://gtexportal.org/home/) (GTEx Consortium, 2020). The GTEx dataset encompasses information from 838 donors and 17,382 samples derived from 52 tissues and two cell lines (GTEx Consortium, 2020). This comprehensive resource enabled the investigation of gene expression across diverse tissues, facilitating insights into the role of specific genes in AF pathogenesis.

### AF outcome datasets

Summary-level data for AF were obtained from the UK Biobank study (Zhou et al., 2020) and FinnGen study (Kurki et al., 2023). Statistics of genetic associations with AF in the UK Biobank were extracted from a GWAS conducted by the Lee Lab. The AF diagnoses were defined according to the International Classification of Diseases, 9th Revision (ICD-9) and ICD-10. In total, there were 60,620 cases and 970,216 controls for AF (Nielsen et al., 2018). Summary-level data of genetic associations with AF were also obtained from the publicly available R10 data release of the FinnGen study. The diagnosis of AF was based on the ICD codes and confirmed by the Social Insurance Institution codes, which a total of 50,743 cases and 210,652 controls for AF, respectively (Nielsen et al., 2018). The discovery stage of the research utilized the UK Biobank study, while the replication stage involved the utilization of data from the FinnGen study.

### Summary-data-based MR analysis

Summary-data-based Mendelian randomization (SMR) was utilized to assess the association between mitochondria-related genes methylation, expression, and protein abundance and the risk of AF (Zhu et al., 2016). SMR offers enhanced statistical power compared to conventional MR analysis when exposure and outcome data are available from independent samples with large sample sizes, particularly based on top associated cis-QTL (Zhu et al., 2016). The selection of top associated cis-QTL involved considering a window centered around the corresponding gene (±1000 kb) and passing a significance threshold of 5.0 × 10^−8^. SNPs with allele frequency differences larger than the specified threshold (set as 0.2 in this study) between any pairwise datasets, including the LD reference sample, the QTL summary data, and the outcome summary data, were excluded. The Heterogeneity in Dependent Instrument (HEIDI) test was employed to discern pleiotropy from linkage, with PHEIDI <0.01 indicating likely pleiotropy and leading to exclusion from the analysis. Both SMR and HEIDI tests were conducted using the SMR software tool (SMR v1.3.1). The resulting *p*-values were adjusted to control the false discovery rate (FDR) at ɑ = 0.05 using the Benjamini–Hochberg method. Associations meeting the criteria of FDR-corrected *p*-value <0.05 and P-HEIDI >0.01 were subjected to colocalization analysis (Chen et al., 2024).

### Colocalization analysis

Colocalization analyses was employed using the coloc R package (version 5.2.3) to identify shared causal variants between AF and mitochondria-related mQTLs, eQTLs, or pQTLs (Giambartolomei et al., 2014). In colocalization analysis, five hypotheses are considered: 1) no causal variants for either trait (H0); 2) a causal variant for gene expression only (H1); 3) a causal variant for disease risk only (H2); 4) distinct causal variants for both traits (H3); and 5) the same shared causal variant for both traits (H4). For colocalization of pQTL-GWAS, eQTL-GWAS, and mQTL-GWAS, the colocalization region windows were set at ±1000 kb, ±1000 kb, and ±500 kb, respectively, based on published articles. The prior probabilities that the causal variants are associated with only trait 1, only trait 2 (AF), and both are respectively set at 1.0 × 10^−4^, 1.0 × 10^−4^, and 1.0 × 10^−5^. A posterior probability of H4 (PPH4) >0.70 was considered supporting evidence of colocalization, with its cutoff corresponding to an FDR of <0.05, strengthening the evidence for a causal relationship analysis (Li et al., 2023; Chen et al., 2024).

### Ethics

All summary statistics used in the MR analysis were generated from previous studies and all original studies were ethically approved and individually consented.

## Results

### Mitochondria-related genes methylation and AF

**FIGURE 2:**
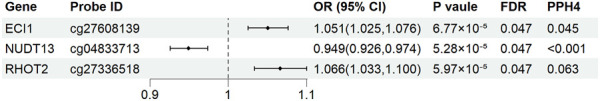
Genetically predicted mitochondria-related genes methylation associated with the risk of atrial fibrillation in Mendelian randomization analysis. OR, odds ratio; CI, confidence interval; FDR, false discovery rate; PPH4, posterior probability of H4.

Results for causal effects of mitochondria-related genes methylation on AF are showed in Figure 2. After the removal of associations with P-HEIDI <0.01, a total of 305 CpG sites near 132 unique genes passed the marginal significance (*p* < 0.05) (Supplementary Table S2). After correction for multiple testing (FDR<0.05), 3 CpG sites near 3 unique genes were identified (Figure 2). Of the 3 identified signals, one SD increase in genetically predicted NUDT13 methylation at cg04833713 was associated with a decreased risk of AF (OR 0.95, 95% confidence interval [CI] 0.93-0.97), whereas one SD increase in genetically predicted ECI1 methylation at cg27608139 and one SD increase in genetically predicted RHOT2 methylation at cg27336518 were associated with increased risk of AF (OR 1.05, 95% CI 1.03–1.08; OR 1.07, 95% CI 1.03–1.10, respectively). Among these identified CpG sites, cg27608139 near ECI1 were replicated in FinnGen (OR 1.04, 95% CI 1.01-1.07m Supplementary Table S3). Unfortunately, no genes were found to be supported by strong evidence of colocalization (PPH4 > 0.70) among the three identified signals.

### Mitochondria-related gene expression and AF

**FIGURE 3:**
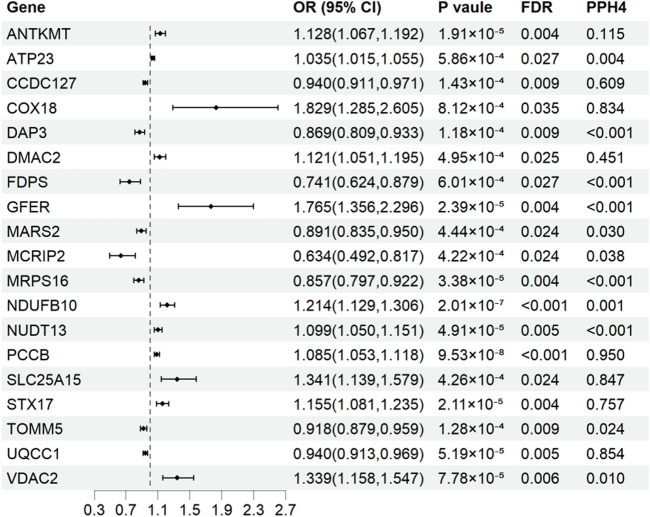
Associations of genetically predicted mitochondria-related genes expression with atrial fibrillation in Mendelian randomization analysis. OR, odds ratio; CI, confidence interval; FDR, false discovery rate; PPH4, posterior probability of H4.

**FIGURE 4:**
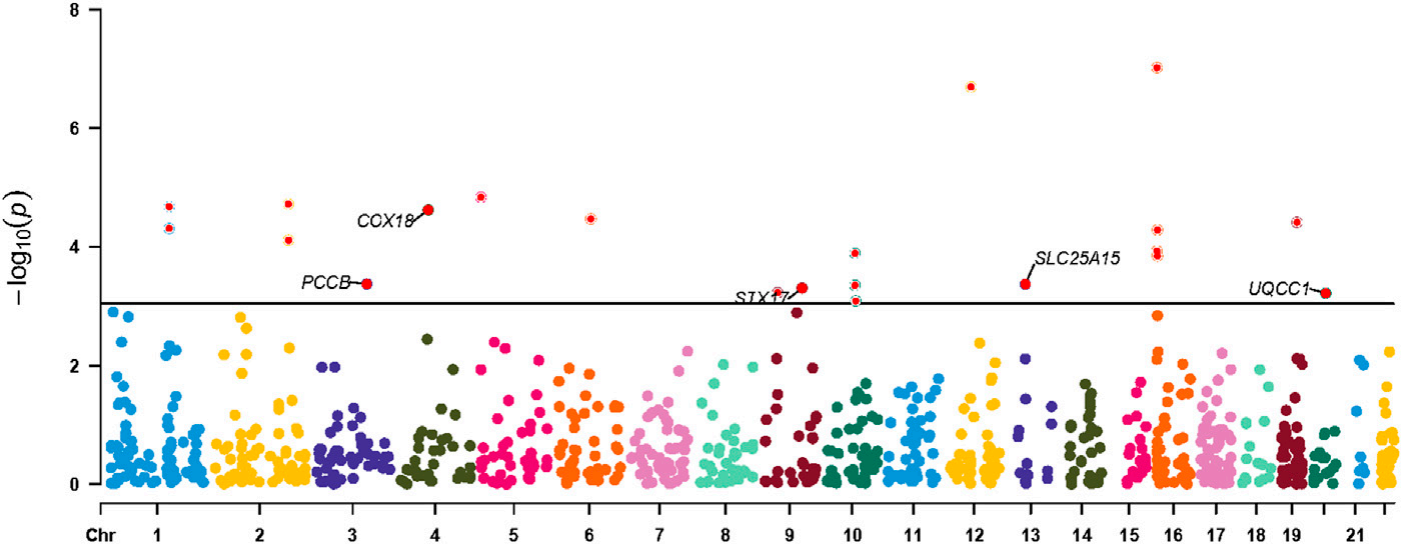
Manhattan plot for associations between mitochondria-related genes molecular features and the risk of atrial fibrillation. Only genes with PPH4 > 0.70 in expression levels were labeled. PPH4, posterior probability of H4.

Results for causal effects of mitochondria-related gene expression on AF were shown in Figure 3. In total, 116 associations were identified to be associated with AF at the nominally significant level (*p* < 0.05, Supplementary Table S4). After multiple testing correction and colocalization analysis, genetically predicted increased expression levels of PCCB (OR 1.09, 95% CI 1.05–1.12; PPH4 = 0.95), COX18 (OR 1.83, 95% CI 1.29–2.60; PPH4 = 0.83), SLC25A15 (OR 1.34, 95% CI 1.14-1.58; PPH4 = 0.85), and STX17 (OR 1.16, 95% CI 1.08–1.24; PPH4 = 0.76) were associated with increased risk of AF (Figures 3, 4). Conversely, genetically predicted increased levels expression of UQCC1 (OR 0.94, 95% CI 0.91–0.97) were associated with a decrease in AF risk (Figures 3, 4). Associations for PCCB and STX17 were all replicated in FinnGen, of which the genetically predicted high level expressions were positively associated with AF risk (OR 1.05, 95% CI 1.01–1.10; OR 1.16, 95% CI 1.06–1.27, respectively, Supplementary Table S5).

### Mitochondria-related gene encoded protein and the risk of AF

A total of 13 proteins encoded by mitochondria-related genes were independently associated with an increased risk of AF at a *p* < 0.05 level. These genes included PARK7, ACP6, ECI2, GUK1, of which the genetically predicted high levels were inversely associated with AF risk; and NME4, NUDT2, PRDX6, MMAB, FAM213A, NT5M, ACAA1, GSR, and DNAJC30, of which the genetically predicted high levels were positively associated with AF risk (Supplementary Table S6). However, after correction for multiple testing, none of the protein levels remained significant in association with the risk of AF.

### Tissue-specific validation

**FIGURE 5:**
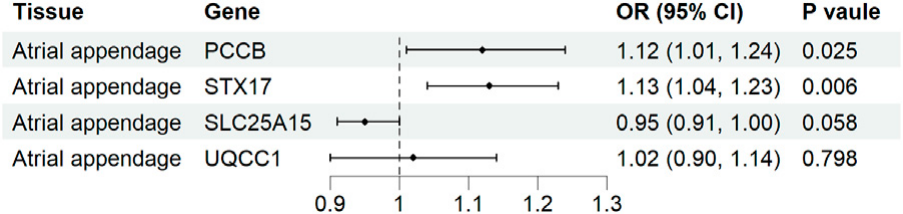
Association of tissue-specific mitochondria-related genes expression with atrial fibrillation. CI, confidence interval; OR, odds ratio.

Causal associations of the expression of identified genes with AF were further explored in atrial tissue (Supplementary Table S8). Genetically predicted expression levels of PCCB and STX17 were associated with increases in AF risk in atrial appendage (OR 1.12, 95% CI 1.01-1.24, *p* = 0.025; OR 1.13, 95% CI 1.04-1.23, *p* = 0.006, respectively). In contrast, genetically predicted expression levels of SLC25A15 and UQCC1 were not associated with AF risk in atrial appendage tissue (OR 0.95, 95% CI 0.91–1.00, *p* = 0.058, and OR 1.02, 95% CI 0.90-1.14, *p* = 0.798, respectively) (Figure 5). The analysis did not include COX18 gene because the outcome data was unavailable.

## Discussion

Proteome-wide MR analysis was utilized in previous studies to identify potential druggable proteins for AF (Davies et al., 2018; Ning et al., 2023). While proteins are the primary executors of biological functions, circulating eQTLs remain crucial for drug-target screening due to their detectability and ease of intervention. In this study, we performed SMR and colocalization analyses to explore the associations between genetically predicted methylation, expression, and protein abundance levels of mitochondria-related genes and the risk of AF. The results of this study indicated that five mitochondria-related genes play a role in the pathogenesis and development of AF, with PCCB, COX18, SLC25A15, and STX17 being positively involved, and UQCCI being negatively involved. Genes PCCB and STX17 were identified as putatively associated with AF risk after tissue-specific validation at transcriptional levels.

PCCB is a beta subunits of the biotin-dependent propionyl-CoA carboxylase (PCC), a mitochondrial enzyme involved in the catabolism of odd chain fatty acids, branched-chain amino acids isoleucine, threonine, methionine and valine. PCCB deficiency causes propionic acidemia (PA), resulting in the accumulation of propionic acid metabolites, and dysfunction in the respiratory chain and urea cycle pathways (Wongkittichote et al., 2017). Patients with PA may exhibit cardiac phenotypes, such as ventricular arrhythmias, long QT syndrome, and dilated cardiomyopathy (Kovacevic et al., 2020; Zhang et al., 2023). In PA mouse models, cardiomyocytes showed elevated ROS levels and an increase in Ca^2+^ sparks, Ca^2+^ waves and spontaneous Ca^2+^ transients, leading to cardiac dysfunction and arrhythmias (Tamayo et al., 2020). As far as we best known, the disease phenotypes resulting from increased PCCB expression have not been explored. In this study, increased PCCB expression was found to be associated with a higher risk of AF, as confirmed by MR and colocalization analysis, and was verified by multiple databases. Elevated PCCB expression may lead to accumulation of the metabolic byproduct methylmalonyl-CoA, which is subsequently converted into succinyl-CoA, a critical intermediate in the tricarboxylic acid cycle (Fernie et al., 2004). Disruptions in this cycle may lead to mitochondrial energy metabolism disturbances and increased production of ATP and ROS (Murphy, 2009). ROS contribute to the pathogenesis of AF by inducing oxidative stress, which drives electrical, structural, and molecular remodelings of atrial tissue. Further studies are needed to understand the underlying mechanisms in depth and to provide novel targets and strategies for AF prevention and treatment.

STX17 is a soluble N-ethylmaleimide-sensitive factor-attachment protein receptors (SNARE) of the autophagosome involved in autophagy through the direct control of autophagosome membrane fusion with the lysosome membrane (Sugo et al., 2018). Increased STX17 expression may affect cellular homeostasis by enhancing, autophagic flux, potentially leading to excessive degradation of cellular components and turnover of organelles (Klionsky et al., 2021). Dysregulations of STX17-mediated autophagy could disrupt the balance between autophagy and cellular processes, leading to accumulation of damaged proteins and organelles, causing cellular dysfunction and oxidative stress, ultimately promoting pathological processes such as fibrosis and inflammation (Sciarretta et al., 2018; Shen et al., 2021). Moreover, elevated STX17 expression can lead to mitochondrial Ca^2+^ overload and increased mitochondrial ROS, resulting in mitochondrial dysfunction and cardiac impairment (Xu et al., 2023). Calcium overload can impair the mitochondrial respiratory chain function, leading to a decrease in ATP production and an increase in generation of ROS (Walkon et al., 2022). Furthermore, Ca^2+^ overload can disrupt mitochondrial permeability transition pore (mPTP) opening, leading to mitochondrial membrane depolarization and release of pro-apoptotic factors (Luongo et al., 2017). This can trigger apoptotic pathways and contribute to cardiomyocyte death, further compromising cardiac function and promoting atrial fibrillation. Through these processes, elevated STX17 expression may contribute to atrial fibrillation, but its actual role and mechanism still need further in-depth study.

Notably, there are three genes, i.e., COX18, UQCC1, and SLC25A15, were identified through SMR and colorization analysis, but tissue-specific evaluation did not support their casual relations with AF. COX18 is involved in cytochrome c oxidase (COX) assembly, which is a key enzyme in the mitochondrial respiratory chain (Bourens and Barrientos, 2017). Specifically, COX18 plays a role in the biogenesis of COX, which is responsible for the final step of electron transfer in the mitochondrial respiratory chain, converting molecular oxygen to water. The UQCC1 gene encodes a protein called ubiquinol-cytochrome c reductase complex assembly factor 1, which is involved in the assembly and function of the mitochondrial respiratory chain complex III (Tucker et al., 2013). Dysfunction of the mitochondrial respiratory chain leads to ROS imbalance and impaired ATP synthesis, which were implicated in the AF development.

The SLC25A15 gene encodes a mitochondrial ornithine transporter protein, also known as the mitochondrial ornithine carrier 1 (ORC1). This transporter plays a crucial role in the urea cycle (Tessa et al., 2009), which is responsible for removing excess nitrogen from the body by converting ammonia into urea in the liver. Specifically, the SLC25A15 protein facilitates the transport of ornithine, an amino acid involved in the urea cycle, across the mitochondrial inner membrane. Mutations in the SLC25A15 gene can lead to ornithine translocase deficiency, a rare inherited metabolic disorder characterized by hyperammonemia and impaired ureagenesis. Hyperammonemia might result in mitochondrial dysfunction and contribute to cellular energy metabolism disturbances and oxidative stress, both of them are implicated in the pathogenesis of AF.

A strength of this study is that a comprehensive MR analysis was performed between mitochondrial dysfunction, characterized by genetic predisposition in all known mitochondria-related genes, and their causal relationships with AF, thus avoiding selection bias and might be able to address the mitochondrial dysfunction directly. Moreover, MR and colocalization analysis were utilized together to analyze mitochondria-related genes variants as causal effects of mitochondria-related genes methylation, expression and protein abundance (Li et al., 2023). Besides, the consistency of results in this study spanned multiple datasets and provided additional support for the major findings.

The limitations of this study include the followings: The causal relationship between mitochondria-related genes and the risk of AF at the methylation and protein levels were not fully explored due to the limited coverage of mitochondria-related genes in the pQTL dataset and mQTL databases currently available for SMR analysis. Although tissue-specific testing of candidate genes were performed using tissue-derived gene databases, the genetic data from blood in circulation may not fully reflect the situation of mitochondria genes in the myocardium. The mitochondrial genome-wide associated genetic variants in this study mainly laid on the mitochondria-related nuclear genome because a mitochondrial genome-specific QTL dataset and GWAS dataset on mitochondrial dysfunction were yet unavailable. Further investigation on the direct causal relationship between mitochondrial proteins and AF risk is warranted.

## Conclusion

The present MR study delves into the potential causal connections of mitochondria-related genes methylation, expression, protein abundance with AF and demonstrates the importance of mitochondria-related genes, specifically PCCB and STX17, dysfunction in the pathogenesis of AF. The identified putative genes may be functioned as potential pharmacological targets for treatment and prevention of AF.

## Data Availability

The original contributions presented in the study are included in the article/Supplementary Material, further inquiries can be directed to the corresponding author.
